# Co-crystallization of *N*′-benzyl­idene­pyridine-4-carbohydrazide and benzoic acid *via* autoxidation of benzaldehyde

**DOI:** 10.1107/S2056989023005698

**Published:** 2023-07-04

**Authors:** Itumeleng B. Setshedi, Andreas Lemmerer, Mark G. Smith

**Affiliations:** a University of South Africa, Department of Life Science, Unisa Science Campus, 28 Pioneer Avenue, Florida, Roodepoort, Gauteng, South Africa; b Molecular Sciences Institute, School of Chemistry, University of the Witwatersrand, Johannesburg, Gauteng, South Africa; c University of South Africa, Chemistry Department, Unisa Science Campus, 28 Pioneer Avenue, Florida, Roodepoort, Gauteng, South Africa; Universidade de Sâo Paulo, Brazil

**Keywords:** crystal structure, autoxidation, benzoic acid, isoniazid

## Abstract

The 1:1 co-crystal *N*′-[(2-methyl­phen­yl)methyl­idene]pyridine-4-carbohydrazide–benzoic acid (1/1), C_13_H_11_N_3_O·C_7_H_6_O_2_, formed unexpectedly after autoxidation of benzaldehyde during the slow evaporation process of a solution of isoniazid in benzaldehyde. The original intent of the synthesis was to modify isoniazid with benzaldehyde and crystallize the product in order to improve efficacy against *Mycobacteria* species, but benzoic acid formed spontaneously and co-crystallized with the intended product, *N*′-benzyl­idene­pyridine-4-carbohydrazide.

## Abstract 2

The 1:1 co-crystal *N*′-[(2-methyl­phen­yl)methyl­idene]pyridine-4-carbohydrazide–benzoic acid (1/1) formed unexpectedly after autoxidation of benzaldehyde during the slow evaporation process of a solution of isoniazid in benzaldehyde. The original intent of the synthesis was to modify isoniazid with benzaldehyde and crystallize the product in order to improve efficacy against *Mycobacteria* species, but benzoic acid formed spontaneously and co-crystallized with the intended product, *N*′-benzyl­idene­pyridine-4-carbohydrazide.

## 1. Chemical context

Mycobacterial infections are a historic tribulation to mankind, and are managed with an array of drugs ranging from natural to synthetic derivatives that possess anti­microbial properties. However, these strategies have failed over time due to the emergence of resistant *mycobacteria* (Cully, 2014 ▸). A number of constituents such as isoniazid (INH) have been modified to try and curb the scourge of tuberculosis (Cully, 2014 ▸). Some of the resulting modified INH derivatives have been shown to render the active pharmaceutical ingredients (API) a lot more active to the circulating resistant strains of TB (Hearn & Cynamon, 2004 ▸; Suarez *et al.*, 2009 ▸). It was for this reason that the covalent modification of API’s was adopted to synthesize new analogues by modifying the NH_2_ group of the hydrazide moiety of INH (Smith *et al.*, 2015 ▸), believed to assist in the evasion of the N-aryl­amino­acetyl transferases, an enzyme capable of reducing the efficacy of INH in particular, by acetyl­ating the NH_2_ position, thus ultimately preventing its reaction with nicotinamide adenine dinucleotide (NADH) (Vishweshwar *et al.*, 2006 ▸; Smith *et al.*, 2015 ▸).

**Figure 1:**
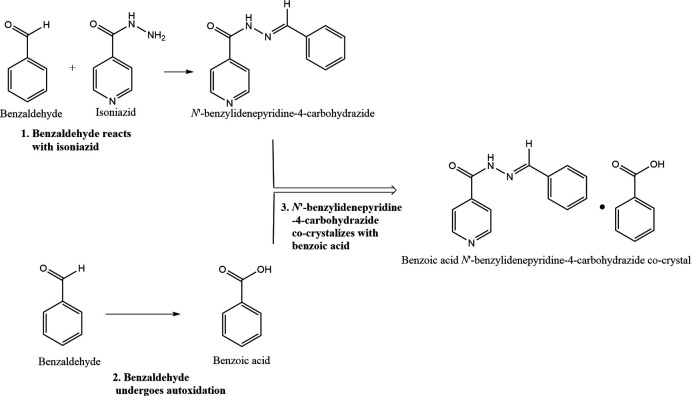
Modification and autoxidative co-crystallization.

Benzaldehyde is known to undergo autoxidation resulting in the formation of benzoic acid. The formation of benzoic acid occurs when benzaldehyde is exposed to air at room temperature (293 K) where the rate of the reaction is increased by the presence of a catalyst. However, this phenomenon can occur spontaneously without a catalyst over a prolonged period (Sankar *et al.*, 2014 ▸). The synthesis of this co-crystal was inter­esting as there were three separate processes that took place within the reaction mixture to create the final product. Firstly, benzaldehyde reacted with isoniazid to form *N′*-benzyl­idene­pyridine-4-carbohydrazide. Secondly, excess benzaldehyde spontaneously autoxidized to form benzoic acid as described above (no benzoic acid was added to the reaction mixture). Lastly, the carbohydrazide moiety co-crystallized with the benzoic acid (as shown in Fig. 1 ▸) to form the product, *N*′-[(2-methyl­phen­yl)methyl­idene]pyridine-4-carbohydrazide–benzoic acid (1/1). 
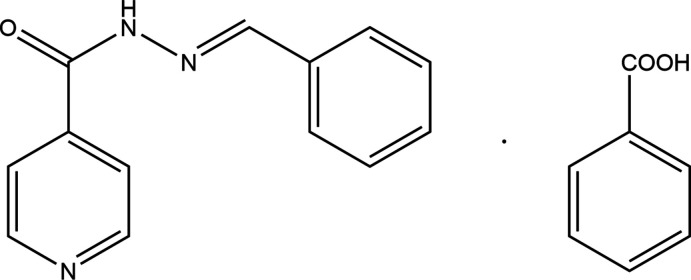


## 2. Structural commentary

**Figure 2:**
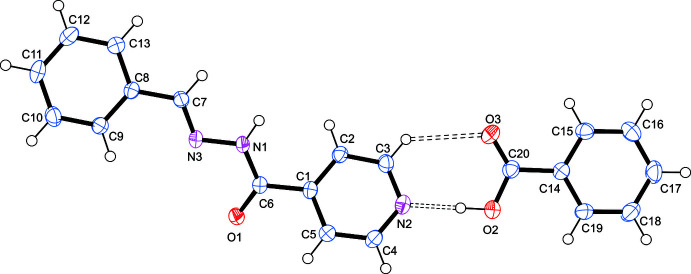
Asymmetric unit of *N*′-[(2-methyl­phen­yl)methyl­idene]pyridine-4-carbohydrazide–benzoic acid (1/1).

The asymmetric unit contains one mol­ecule of N′-benzyl­idene­pyridine-4-carbohydrazide (C_13_H_11_N_3_O_1_·C_7_H_6_O_2_) and one mol­ecule of benzoic acid (as shown in Fig. 2 ▸). This co-crystal crystallizes in the *Pbca* space group. The benzoic acid mol­ecule lies in the plane of the pyridine ring of the benzyl­idene derivative. All bond lengths and angles are normal.

## 3. Supra­molecular features

**Figure 3:**
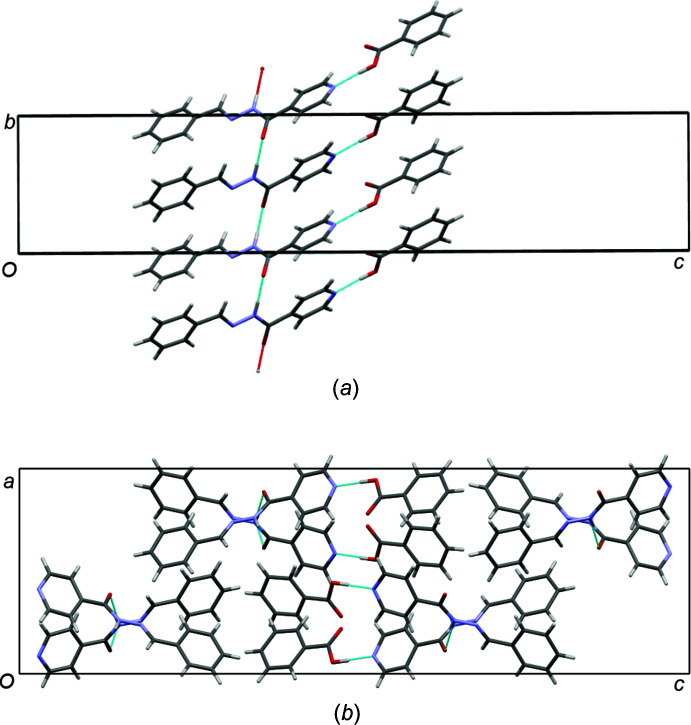
(*a*) View of the strong hydrogen bonds that link the carbohydrazide mol­ecules in a chain, and the benzoic acid mol­ecules to the carbohydrazide forming a ribbon. (*b*) Packing diagram along the *b* axis.

Each carbohydrazide moiety is hydrogen bonded by a strong O2—H2⋯N2 hydrogen bond (Table 1 ▸) to a benzoic acid mol­ecule to form a co-crystal. This inter­action is supported by a weaker C—H⋯O hydrogen bond that stabilizes the co-planar arrangement of the carb­oxy­lic acid moiety and the pyridine ring. The graph-set notation for this would be 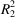 (7) (Bernstein *et al.*, 1995 ▸), and is observed in other isoniazid co-crystals (Lemmerer *et al.*, 2010 ▸) (Fig. 2 ▸). This co-crystal is another example of the robust carb­oxy­lic acid⋯pyridine heterosynthon (Shattock *et al.*, 2008 ▸; Aakeröy *et al.*, 2007 ▸). Each carbohydrazide moiety is also hydrogen bonded *via* its N1—H1 donor to the carbonyl oxygen (O1) acceptor of an adjacent carbohydrazide moiety. This results in a mono-periodic hydrogen-bonded chain along the *b*-axis direction, with graph-set notation *C*(4). Overall, the combined carbohydrazide moiety with the benzoic acid forms a ribbon motif (as shown in Fig. 3 ▸ *a*). Viewed along the *b*-axis, the ribbons forms a X-shaped motif seen in other carbohydrazide moieties (Hean *et al.* 2018 ▸) (Fig. 3 ▸ *b*).

## 4. Database survey

**Figure 4:**
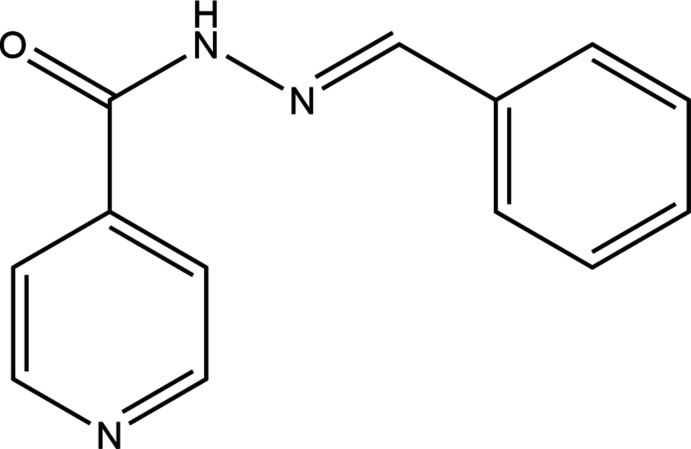
Benzyl­idene derivative of isoniazid.

ConQuest (Bruno *et al.*, 2002 ▸), Version 2022.1.0 of the CSD (Groom *et al.*, 2016 ▸) was used for the database survey, where only one similar structure was found. The survey consisted of structures consisting of isoniazid that had been modified with benzaldehyde and may have either a co-former or solvent mol­ecule in the crystal structure. The structure of the anhydrous benzyl­idene derivative, *N*′-[(2-methyl­phen­yl)methyl­idene]pyridine-4-carbohydrazide (as shown in Fig. 4 ▸), formed from the reaction of isoniazid and benzaldehyde, was reported by Wardell *et al.*, (2007 ▸) (YIQDEI). Several structures have been reported where substituted benzaldehyde reacted with isoniazid, for example, three polymorphs of the 4-methyl­benzyl­idene derivative (WOGGOR, WOGGOR01 and WOGGUX) were reported by Purushothaman *et al.* (2019 ▸) and in 2016, Almeida and colleagues published the structure of a hydrate of the same 4-methyl­benzyl­idene derivative (OLECOZ; Pereira Almeida *et al.*, 2016 ▸). However, there has not been any co-crystal of the benzyl­idene derivative (Fig. 4 ▸) reported in the literature to date.

## 5. Synthesis and crystallization

All reagents were commercially sourced and used without further purification. 1.00 g of isonicotinic acid hydrazide (isoniazid) (7.29 mmol) were dissolved in 15 ml of benzaldehyde in a 50 ml amber Schott bottle. The mixture was placed on a stirring heating block and heated to 333 K while stirring with a magnetic stirrer bar. Once the isoniazid had completely dissolved, the lid was tightly sealed. The solution was then allowed to react for 24 h. To maintain the temperature throughout the duration of the experiment, the amber Schott bottle was covered with an inverted round glass evaporation dish. After 24 h, the solution was allowed to cool to ambient temperature. The stirrer bar was retrieved and the sample was left to evaporate slowly for 6 weeks at ambient temperature without a lid. Over the 6 weeks, the temperature in the laboratory fluctuated between 298 and 300 K. Due to the fact that benzaldehyde evaporates extremely slowly, the Schott bottle was placed in the laminar flow biohazard safety level 2 cabinet to facilitate evaporation. Crystals (colourless blocks) started forming on the rim on the outside of the bottle as the benzaldehyde evaporated. One of these crystals was sampled for XRD analysis.

## 6. Refinement

Crystal data, data collection and structure refinement details are summarized in Table 2 ▸. C-bound H atoms were first located in the difference map, then positioned geometrically and allowed to ride on their respective parent atoms, with thermal displacement parameters 1.2 times of the parent C atom. The coordinates and isotropic displacement parameters of the O and N-bound H atoms involved in hydrogen-bonding inter­actions (H1 and H2) were allowed to refine freely.

## Supplementary Material

Crystal structure: contains datablock(s) global, I. DOI: 10.1107/S2056989023005698/ex2072sup1.cif

Structure factors: contains datablock(s) I. DOI: 10.1107/S2056989023005698/ex2072Isup3.hkl

Supporting information file. DOI: 10.1107/S2056989023005698/ex2072Isup5.mol

Supporting information file. DOI: 10.1107/S2056989023005698/ex2072Isup4.cml

CCDC reference: 2250754

Additional supporting information:  crystallographic information; 3D view; checkCIF report

## Figures and Tables

**Table 1:** Hydrogen-bond geometry (Å, °)

*D*—H⋯*A*	*D*—H	H⋯*A*	*D*⋯*A*	*D*—H⋯*A*
N1—H1⋯O1^i^	0.89 (2)	2.02 (2)	2.8981 (17)	171 (2)
O2—H2⋯N2	0.95 (2)	1.72 (3)	2.6693 (17)	176 (2)
C3—H3⋯O3	0.95	2.53	3.236 (2)	131

Symmetry code: (i) 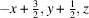 .

**Table 2:** Experimental details

Crystal data	
Chemical formula	C_13_H_11_N_3_O·C_7_H_6_O_2_
*M*_r_	347.36
Crystal system, space group	Orthorhombic, *P* *b* *c* *a*
Temperature (K)	173
*a*, *b*, *c* (Å)	11.7044 (17), 7.8531 (10), 38.253 (5)
*V* (Å^3^)	3516.1 (8)
*Z*	8
Radiation type	Mo *K*α
μ (mm^−1^)	0.09
Crystal size (mm)	0.30 × 0.22 × 0.16

Data collection	
Diffractometer	Bruker D8 Venture Microfocus with Photon III CCD area-detector
Absorption correction	Multi-scan (*SADABS*; Krause *et al.*, 2015 ▸)
*T*_min_, *T*_max_	0.709, 0.746
No. of measured, independent and observed [*I* > 2σ(*I*)] reflections	73794, 4239, 3902
*R*_int_	0.036
(sin θ/λ)_max_ (Å^−1^)	0.660

Refinement	
*R*[*F* ^2^ > 2σ(*F* ^2^)], *wR*(*F* ^2^), *S*	0.054, 0.112, 1.18
No. of reflections	4239
No. of parameters	243
H-atom treatment	H atoms treated by a mixture of independent and constrained refinement
Δρ_max_, Δρ_min_ (e Å^−3^)	0.33, −0.21

Computer programs: *APEX3*, *SAINT-Plus* and *XPREP* (Bruker 2016 ▸), *SHELXT2014* (Sheldrick, 2015*a* ▸), *SHELXL2019/2* (Sheldrick, 2015*b* ▸) and *ORTEP-3 for Windows* and *WinGX* (Farrugia, 2012 ▸).

## supplementary crystallographic information

### Crystal data

**Table:**

C_13_H_11_N_3_O·C_7_H_6_O_2_	*D*_x_ = 1.312 Mg m^−^^3^
*M**_r_* = 347.36	Mo *K*α radiation, λ = 0.71073 Å
Orthorhombic, *P**b**c**a*	Cell parameters from 9857 reflections
*a* = 11.7044 (17) Å	θ = 2.8–28.1°
*b* = 7.8531 (10) Å	µ = 0.09 mm^−^^1^
*c* = 38.253 (5) Å	*T* = 173 K
*V* = 3516.1 (8) Å^3^	Block, colourless
*Z* = 8	0.30 × 0.22 × 0.16 mm
*F*(000) = 1456	

### Data collection

**Table:**

Bruker D8 Venture Microfocus with Photon III CCD area-detector diffractometer	3902 reflections with *I* > 2σ(*I*)
Graphite monochromator	*R*_int_ = 0.036
ω scans	θ_max_ = 28.0°, θ_min_ = 3.2°
Absorption correction: multi-scan (SADABS; Krause *et al.*, 2015)	*h* = −15→15
*T*_min_ = 0.709, *T*_max_ = 0.746	*k* = −8→10
73794 measured reflections	*l* = −49→50
4239 independent reflections	

### Refinement

**Table:**

Refinement on *F*^2^	Primary atom site location: dual
Least-squares matrix: full	Secondary atom site location: dual
*R*[*F*^2^ > 2σ(*F*^2^)] = 0.054	Hydrogen site location: mixed
*wR*(*F*^2^) = 0.112	H atoms treated by a mixture of independent and constrained refinement
*S* = 1.18	*w* = 1/[σ^2^(*F*_o_^2^) + (0.0274*P*)^2^ + 2.3245*P*] where *P* = (*F*_o_^2^ + 2*F*_c_^2^)/3
4239 reflections	(Δ/σ)_max_ = 0.001
243 parameters	Δρ_max_ = 0.33 e Å^−^^3^
0 restraints	Δρ_min_ = −0.21 e Å^−^^3^
0 constraints	

### Special details

**Table:**

Experimental. Absorption corrections were made using the program SADABS (Sheldrick, 1996)
Geometry. All esds (except the esd in the dihedral angle between two l.s. planes) are estimated using the full covariance matrix. The cell esds are taken into account individually in the estimation of esds in distances, angles and torsion angles; correlations between esds in cell parameters are only used when they are defined by crystal symmetry. An approximate (isotropic) treatment of cell esds is used for estimating esds involving l.s. planes.
Refinement. The crystal structure was solved through direct methods using *SHELXT*. Non-hydrogen atoms were first refined isotropically followed by anisotropic refinement by full matrix least-squares calculations based on *F*^2^ using *SHELXL*.

### Fractional atomic coordinates and isotropic or equivalent isotropic displacement parameters (Å^2^)

**Table:**

	*x*	*y*	*z*	*U*_iso_*/*U*_eq_	
C1	0.63979 (12)	0.05245 (18)	0.40684 (4)	0.0224 (3)	
C2	0.71809 (13)	0.15589 (19)	0.42397 (4)	0.0252 (3)	
H2A	0.79074	0.178612	0.413922	0.03*	
C3	0.68809 (13)	0.2256 (2)	0.45612 (4)	0.0278 (3)	
H3	0.742184	0.295037	0.467926	0.033*	
C4	0.51195 (13)	0.0976 (2)	0.45471 (4)	0.0294 (3)	
H4	0.439948	0.077268	0.465382	0.035*	
C5	0.53502 (13)	0.0207 (2)	0.42278 (4)	0.0273 (3)	
H5	0.48046	−0.052042	0.412045	0.033*	
C6	0.66282 (12)	−0.03373 (18)	0.37245 (4)	0.0217 (3)	
C7	0.83497 (12)	0.04298 (19)	0.29981 (4)	0.0241 (3)	
H7	0.875011	0.138628	0.308817	0.029*	
C8	0.85942 (12)	−0.01869 (18)	0.26424 (4)	0.0225 (3)	
C9	0.78697 (13)	−0.13366 (19)	0.24744 (4)	0.0275 (3)	
H9	0.72116	−0.175227	0.259209	0.033*	
C10	0.81045 (14)	−0.1876 (2)	0.21367 (4)	0.0325 (3)	
H10	0.761097	−0.266749	0.202491	0.039*	
C11	0.90585 (15)	−0.1264 (2)	0.19618 (4)	0.0329 (4)	
H11	0.921511	−0.16284	0.17299	0.039*	
C12	0.97809 (14)	−0.0121 (2)	0.21258 (4)	0.0330 (4)	
H12	1.043564	0.029452	0.200633	0.04*	
C13	0.95532 (13)	0.0423 (2)	0.24649 (4)	0.0288 (3)	
H13	1.005101	0.121063	0.257586	0.035*	
N1	0.73940 (11)	0.04239 (16)	0.35104 (3)	0.0243 (3)	
N2	0.58653 (11)	0.19911 (17)	0.47113 (3)	0.0284 (3)	
N3	0.75993 (11)	−0.03198 (16)	0.31860 (3)	0.0235 (3)	
O1	0.61404 (9)	−0.16790 (13)	0.36487 (3)	0.0264 (2)	
H1	0.7778 (16)	0.136 (3)	0.3562 (5)	0.035 (5)*	
C14	0.62773 (13)	0.54692 (19)	0.57434 (4)	0.0255 (3)	
C15	0.71251 (13)	0.6581 (2)	0.58553 (4)	0.0296 (3)	
H15	0.776244	0.680985	0.570885	0.036*	
C16	0.70449 (15)	0.7358 (2)	0.61798 (4)	0.0359 (4)	
H16	0.762819	0.81133	0.625584	0.043*	
C17	0.61171 (16)	0.7034 (3)	0.63923 (4)	0.0415 (4)	
H17	0.605726	0.757686	0.661344	0.05*	
C18	0.52751 (16)	0.5920 (3)	0.62834 (5)	0.0463 (5)	
H18	0.463906	0.569504	0.643068	0.056*	
C19	0.53547 (14)	0.5127 (2)	0.59597 (4)	0.0365 (4)	
H19	0.477851	0.435326	0.588664	0.044*	
C20	0.63940 (13)	0.4638 (2)	0.53921 (4)	0.0281 (3)	
O2	0.55589 (10)	0.35567 (16)	0.53235 (3)	0.0357 (3)	
O3	0.71911 (11)	0.49208 (17)	0.51992 (3)	0.0428 (3)	
H2	0.565 (2)	0.303 (3)	0.5101 (6)	0.070 (7)*	

### Atomic displacement parameters (Å^2^)

**Table:**

	*U*^11^	*U*^22^	*U*^33^	*U*^12^	*U*^13^	*U*^23^
C1	0.0269 (7)	0.0212 (6)	0.0192 (6)	0.0038 (6)	−0.0001 (5)	0.0006 (5)
C2	0.0264 (7)	0.0278 (7)	0.0212 (6)	−0.0004 (6)	0.0014 (5)	0.0009 (6)
C3	0.0313 (8)	0.0302 (8)	0.0220 (7)	−0.0009 (6)	−0.0012 (6)	−0.0021 (6)
C4	0.0280 (7)	0.0339 (8)	0.0261 (7)	0.0010 (6)	0.0051 (6)	0.0002 (6)
C5	0.0266 (7)	0.0280 (7)	0.0273 (7)	−0.0013 (6)	0.0017 (6)	−0.0017 (6)
C6	0.0229 (7)	0.0218 (6)	0.0205 (6)	0.0042 (5)	−0.0018 (5)	−0.0001 (5)
C7	0.0262 (7)	0.0235 (7)	0.0226 (7)	−0.0011 (6)	−0.0010 (5)	−0.0012 (5)
C8	0.0258 (7)	0.0214 (6)	0.0203 (6)	0.0027 (6)	0.0007 (5)	0.0017 (5)
C9	0.0296 (7)	0.0304 (7)	0.0226 (7)	−0.0043 (6)	0.0001 (6)	0.0015 (6)
C10	0.0383 (8)	0.0353 (8)	0.0240 (7)	−0.0029 (7)	−0.0047 (6)	−0.0032 (6)
C11	0.0403 (9)	0.0392 (9)	0.0192 (7)	0.0088 (7)	0.0023 (6)	−0.0003 (6)
C12	0.0298 (8)	0.0415 (9)	0.0276 (8)	0.0025 (7)	0.0075 (6)	0.0043 (7)
C13	0.0267 (7)	0.0317 (8)	0.0279 (7)	−0.0022 (6)	0.0018 (6)	−0.0009 (6)
N1	0.0289 (6)	0.0238 (6)	0.0201 (5)	−0.0027 (5)	0.0019 (5)	−0.0051 (5)
N2	0.0340 (7)	0.0300 (7)	0.0212 (6)	0.0026 (5)	0.0023 (5)	−0.0012 (5)
N3	0.0266 (6)	0.0254 (6)	0.0185 (5)	0.0017 (5)	0.0003 (5)	−0.0031 (5)
O1	0.0295 (5)	0.0244 (5)	0.0252 (5)	−0.0022 (4)	0.0007 (4)	−0.0028 (4)
C14	0.0261 (7)	0.0264 (7)	0.0240 (7)	0.0016 (6)	−0.0001 (6)	0.0018 (6)
C15	0.0294 (7)	0.0300 (8)	0.0294 (7)	−0.0038 (6)	0.0006 (6)	0.0032 (6)
C16	0.0382 (9)	0.0349 (8)	0.0348 (8)	−0.0053 (7)	−0.0086 (7)	−0.0029 (7)
C17	0.0439 (10)	0.0542 (11)	0.0263 (8)	0.0004 (9)	−0.0018 (7)	−0.0125 (8)
C18	0.0338 (9)	0.0726 (14)	0.0324 (9)	−0.0093 (9)	0.0096 (7)	−0.0118 (9)
C19	0.0277 (8)	0.0499 (10)	0.0318 (8)	−0.0110 (7)	0.0037 (6)	−0.0070 (7)
C20	0.0312 (7)	0.0282 (7)	0.0250 (7)	−0.0002 (6)	0.0023 (6)	0.0016 (6)
O2	0.0366 (6)	0.0436 (7)	0.0271 (6)	−0.0091 (5)	0.0055 (5)	−0.0098 (5)
O3	0.0474 (8)	0.0493 (7)	0.0316 (6)	−0.0129 (6)	0.0156 (5)	−0.0054 (6)

### Geometric parameters (Å, º)

**Table:**

C1—C2	1.389 (2)	C11—C12	1.384 (2)
C1—C5	1.392 (2)	C11—H11	0.95
C1—C6	1.5039 (19)	C12—C13	1.391 (2)
C2—C3	1.391 (2)	C12—H12	0.95
C2—H2A	0.95	C13—H13	0.95
C3—N2	1.336 (2)	N1—N3	1.3922 (16)
C3—H3	0.95	N1—H1	0.89 (2)
C4—N2	1.338 (2)	C14—C19	1.387 (2)
C4—C5	1.389 (2)	C14—C15	1.389 (2)
C4—H4	0.95	C14—C20	1.500 (2)
C5—H5	0.95	C15—C16	1.386 (2)
C6—O1	1.2328 (17)	C15—H15	0.95
C6—N1	1.3533 (19)	C16—C17	1.380 (2)
C7—N3	1.2784 (19)	C16—H16	0.95
C7—C8	1.4725 (19)	C17—C18	1.382 (3)
C7—H7	0.95	C17—H17	0.95
C8—C9	1.395 (2)	C18—C19	1.389 (2)
C8—C13	1.397 (2)	C18—H18	0.95
C9—C10	1.387 (2)	C19—H19	0.95
C9—H9	0.95	C20—O3	1.2101 (19)
C10—C11	1.388 (2)	C20—O2	1.3210 (19)
C10—H10	0.95	O2—H2	0.95 (2)
			
C2—C1—C5	118.66 (13)	C11—C12—C13	120.31 (15)
C2—C1—C6	123.89 (13)	C11—C12—H12	119.8
C5—C1—C6	117.41 (13)	C13—C12—H12	119.8
C3—C2—C1	118.72 (14)	C12—C13—C8	120.14 (15)
C3—C2—H2A	120.6	C12—C13—H13	119.9
C1—C2—H2A	120.6	C8—C13—H13	119.9
N2—C3—C2	122.90 (14)	C6—N1—N3	117.93 (12)
N2—C3—H3	118.6	C6—N1—H1	124.6 (12)
C2—C3—H3	118.6	N3—N1—H1	117.5 (12)
N2—C4—C5	123.01 (14)	C3—N2—C4	118.11 (13)
N2—C4—H4	118.5	C7—N3—N1	115.24 (12)
C5—C4—H4	118.5	C19—C14—C15	119.61 (14)
C4—C5—C1	118.57 (14)	C19—C14—C20	121.40 (14)
C4—C5—H5	120.7	C15—C14—C20	118.98 (14)
C1—C5—H5	120.7	C16—C15—C14	120.27 (15)
O1—C6—N1	122.82 (13)	C16—C15—H15	119.9
O1—C6—C1	120.47 (13)	C14—C15—H15	119.9
N1—C6—C1	116.70 (12)	C17—C16—C15	119.97 (16)
N3—C7—C8	120.11 (13)	C17—C16—H16	120
N3—C7—H7	119.9	C15—C16—H16	120
C8—C7—H7	119.9	C16—C17—C18	120.03 (16)
C9—C8—C13	119.10 (13)	C16—C17—H17	120
C9—C8—C7	121.35 (13)	C18—C17—H17	120
C13—C8—C7	119.53 (13)	C17—C18—C19	120.28 (16)
C10—C9—C8	120.42 (14)	C17—C18—H18	119.9
C10—C9—H9	119.8	C19—C18—H18	119.9
C8—C9—H9	119.8	C14—C19—C18	119.84 (16)
C9—C10—C11	120.16 (15)	C14—C19—H19	120.1
C9—C10—H10	119.9	C18—C19—H19	120.1
C11—C10—H10	119.9	O3—C20—O2	124.57 (15)
C12—C11—C10	119.87 (14)	O3—C20—C14	122.44 (15)
C12—C11—H11	120.1	O2—C20—C14	112.98 (13)
C10—C11—H11	120.1	C20—O2—H2	112.0 (15)
			
C5—C1—C2—C3	−0.9 (2)	C7—C8—C13—C12	−178.61 (14)
C6—C1—C2—C3	−178.49 (13)	O1—C6—N1—N3	2.5 (2)
C1—C2—C3—N2	−0.8 (2)	C1—C6—N1—N3	−178.20 (11)
N2—C4—C5—C1	−1.0 (2)	C2—C3—N2—C4	1.6 (2)
C2—C1—C5—C4	1.7 (2)	C5—C4—N2—C3	−0.7 (2)
C6—C1—C5—C4	179.49 (13)	C8—C7—N3—N1	−176.72 (12)
C2—C1—C6—O1	152.30 (14)	C6—N1—N3—C7	−178.41 (13)
C5—C1—C6—O1	−25.3 (2)	C19—C14—C15—C16	−0.6 (2)
C2—C1—C6—N1	−27.0 (2)	C20—C14—C15—C16	−179.49 (15)
C5—C1—C6—N1	155.35 (13)	C14—C15—C16—C17	−0.3 (3)
N3—C7—C8—C9	13.8 (2)	C15—C16—C17—C18	0.8 (3)
N3—C7—C8—C13	−167.96 (14)	C16—C17—C18—C19	−0.3 (3)
C13—C8—C9—C10	0.5 (2)	C15—C14—C19—C18	1.1 (3)
C7—C8—C9—C10	178.77 (14)	C20—C14—C19—C18	179.94 (17)
C8—C9—C10—C11	−0.6 (2)	C17—C18—C19—C14	−0.7 (3)
C9—C10—C11—C12	0.5 (3)	C19—C14—C20—O3	179.85 (17)
C10—C11—C12—C13	−0.3 (3)	C15—C14—C20—O3	−1.3 (2)
C11—C12—C13—C8	0.2 (2)	C19—C14—C20—O2	−1.6 (2)
C9—C8—C13—C12	−0.3 (2)	C15—C14—C20—O2	177.25 (14)

### Hydrogen-bond geometry (Å, º)

**Table:**

*D*—H···*A*	*D*—H	H···*A*	*D*···*A*	*D*—H···*A*
N1—H1···O1^i^	0.89 (2)	2.02 (2)	2.8981 (17)	171 (2)
O2—H2···N2	0.95 (2)	1.72 (3)	2.6693 (17)	176 (2)
C3—H3···O3	0.95	2.53	3.236 (2)	131

Symmetry code: (i) −*x*+3/2, *y*+1/2, *z*.

## References

[bb1] Aakeröy, C. B., Hussain, I., Forbes, S. & Desper, J. (2007). *CrystEngComm*, **9**, 46–54.

[bb2] Bernstein, J., Davis, R. E., Shimoni, L. & Chang, N.-L. (1995). *Angew. Chem. Int. Ed. Engl.* **34**, 1555–1573.

[bb3] Bruker (2016). *APEX3*, *SAINT-Plus* and *XPREP*. Bruker AXS Inc., Madison, Wisconsin, USA.

[bb4] Bruno, I. J., Cole, J. C., Edgington, P. R., Kessler, M., Macrae, C. F., McCabe, P., Pearson, J. & Taylor, R. (2002). *Acta Cryst.* B**58**, 389–397. 10.1107/s0108768102003324 12037360

[bb5] Cully, M. (2014). *Nat. Rev. Drug Discov.* **13**, 257. https://doi.org/10.1038/nrd4287

[bb6] Farrugia, L. J. (2012). *J. Appl. Cryst.* **45**, 849–854.

[bb7] Groom, C. R., Bruno, I. J., Lightfoot, M. P. & Ward, S. C. (2016). *Acta Cryst.* B**72**, 171–179. 10.1107/S2052520616003954 PMC4822653 27048719

[bb8] Hean, D., Michael, J. P. & Lemmerer, A. (2018). *J. Mol. Struct.* **1157**, 693–707.

[bb9] Hearn, M. J. & Cynamon, M. H. (2004). *J. Antimicrob. Chemother.* **53**, 185–191. 10.1093/jac/dkh041 14688045

[bb10] Krause, L., Herbst-Irmer, R., Sheldrick, G. M. & Stalke, D. (2015). *J. Appl. Cryst.* **48**, 3–10. 10.1107/S1600576714022985 PMC4453166 26089746

[bb11] Lemmerer, A., Bernstein, J. & Kahlenberg, V. (2010). *CrystEngComm*, **12**, 2856–2864.

[bb21] Pereira Almeida, W., Paes Koury, I. & Simoni, D. de A. (2016). *IUCrData*, **1**, x160752.

[bb12] Purushothaman, G., Angira, D. & Thiruvenkatam, V. (2019). *J. Mol. Struct.* **1197**, 34–44.

[bb20] Sankar, M., Nowicka, E., Carter, E., Murphy, D., Knight, D., Bethell, D. & Hutchings, G. (2014). *Nat. Commun.* **5**, 3332. https://doi.org/10.1038/ncomms4332 10.1038/ncomms4332 24567108

[bb13] Shattock, T. R., Arora, K. K., Vishweshwar, P. & Zaworotko, M. J. (2008). *Cryst. Growth Des.* **8**, 4533–4545.

[bb14] Sheldrick, G. M. (2015*a*). *Acta Cryst.* A**71**, 3–8.

[bb15] Sheldrick, G. M. (2015*b*). *Acta Cryst.* C**71**, 3–8.

[bb16] Smith, M. G., Forbes, R. P. & Lemmerer, A. (2015). *Cryst. Growth Des.* **15**, 3813–3821.

[bb17] Suarez, J., Ranguelova, K., Jarzecki, A. A., Manzerova, J., Krymov, V., Zhao, X., Yu, S., Metlitsky, L., Gerfen, G. J. & Magliozzo, R. S. (2009). *J. Biol. Chem.* **284**, 7017–7029. 10.1074/jbc.M808106200 PMC2652337 19139099

[bb18] Vishweshwar, P., McMahon, J. A., Bis, J. A. & Zaworotko, M. J. (2006). *J. Pharm. Sci.* **95**, 499–516. 10.1002/jps.20578 16444755

[bb19] Wardell, S. M. S. V., de Souza, M. V. N., Wardell, J. L., Low, J. N. & Glidewell, C. (2007). *Acta Cryst.* B**63**, 879–895. 10.1107/S0108768107036270 18004043

